# Ocular Toxoplasmosis: Advances in *Toxoplasma gondii* Biology, Clinical Manifestations, Diagnostics, and Therapy

**DOI:** 10.3390/pathogens13100898

**Published:** 2024-10-14

**Authors:** Miki Miyagaki, Yuan Zong, Mingming Yang, Jing Zhang, Yaru Zou, Kyoko Ohno-Matsui, Koju Kamoi

**Affiliations:** Department of Ophthalmology & Visual Science, Graduate School of Medical and Dental Sciences, Tokyo Medical and Dental University, Tokyo 113-8510, Japan; miyagakimk@gmail.com (M.M.); zongyuan666@gmail.com (Y.Z.); yangmm-12@outlook.com (M.Y.); zhangj.c@foxmail.com (J.Z.); alicezouyaru519@gmail.com (Y.Z.); k.ohno.oph@tmd.ac.jp (K.O.-M.)

**Keywords:** *Toxoplasma gondii*, Ocular toxoplasmosis, Diagnostic techniques, Pathogenesis research

## Abstract

*Toxoplasma gondii*, an obligate intracellular parasite, is a globally prevalent pathogen capable of infecting a wide range of warm-blooded animals, including humans. Ocular toxoplasmosis (OT), a severe manifestation of *T. gondii* infection, can lead to potentially blinding complications. This comprehensive review delves into the current understanding of *T. gondii* biology, exploring its complex life cycle, diverse transmission routes, and strain diversity. This article provides an in-depth analysis of the clinical manifestations of OT, which can result from both congenital and acquired infections, presenting a spectrum of signs and symptoms. The review examines various diagnostic strategies employed for OT, including clinical examination, multimodal imaging techniques such as fundus fluorescein angiography (FFA), indocyanine green angiography (ICGA), optical coherence tomography (OCT), and optical coherence tomography angiography (OCTA), as well as laboratory tests including serology and molecular methods. Despite extensive research, the specific mechanisms underlying ocular involvement in *T. gondii* infection remain elusive, and current diagnostic options have limitations. Moreover, the treatment of active and recurrent OT remains a challenge. While existing therapies, such as antimicrobial agents and immunosuppressants, can control active infections, they do not offer a definitive cure or completely prevent recurrence. The clinical endpoints for the management of active and recurrent OT are also not yet well-established, and the available treatment methods carry the potential for adverse effects. This article highlights the need for future research to elucidate the pathogenesis of OT, investigate genetic factors influencing susceptibility to infection, and develop more sensitive and specific diagnostic tools. Enhancing global surveillance, implementing robust prevention strategies, and fostering multidisciplinary collaborations will be crucial in reducing the burden of OT and improving patient outcomes. This comprehensive review aims to provide a valuable resource for clinicians, researchers, and policymakers, contributing to a better understanding of *T. gondii* infection and its impact on ocular health.

## 1. Introduction

Toxoplasmosis is a globally prevalent zoonotic disease caused by the protozoan parasite *Toxoplasma gondii* (*T. gondii*) [1,2]. *T. gondii*, an obligate intracellular protozoan parasite belonging to the phylum Apicomplexa, exhibits a complex and unique life cycle. This ubiquitous pathogen can infect a wide range of warm-blooded animals, including humans, which serve as intermediate hosts in its life cycle [3]. It is estimated that over one-third of the world’s population are infected with this parasite [1,4]. Infection rates vary significantly across different regions and populations, influenced by factors such as geography, dietary habits, economic conditions, and sanitation standards [4]. Among women of childbearing age, the prevalence of latent toxoplasmosis ranges from as low as 4% in South Korea to as high as 74% in Ethiopia [1]. Toxoplasmosis transmission occurs through multiple routes, including ingestion of oocyst-contaminated food or water, consumption of undercooked meat containing tissue cysts, and congenital transmission. The latter refers to the primary infection of pregnant women leading to transplacental transmission to the fetus [5]. Upon ingestion, sporozoites released from oocysts or bradyzoites from tissue cysts infect human intestinal epithelial cells. These then rapidly proliferate into tachyzoites, causing cell lysis and dissemination throughout host tissues, which leads to the acute phase of clinical disease. In response to host immune pressure, tachyzoites differentiate into slow-replicating bradyzoites within tissue cysts, establishing a chronic, latent infection. This stage is characterized by limited pathogenicity but lifelong persistence. Immunosuppression can trigger bradyzoite-to-tachyzoite conversion, resulting in reactivation of infection, cellular damage, and clinical manifestations [3]. The three classical clinical forms of toxoplasmosis include ocular toxoplasmosis (OT), congenital toxoplasmosis (CT), and cerebral toxoplasmosis [6].

OT encompasses a spectrum of eye disorders resulting from *T. gondii* infection. The primary manifestations include retinochoroiditis, optic neuritis, and uveitis. Posterior pole retinochoroiditis is the most prevalent form. This condition is characterized by yellowish-white retinal lesions and decreased visual acuity [2,7]. Uveitis encompasses inflammatory conditions affecting the uveal tract, which comprises the iris, ciliary body, and choroid. This intraocular inflammation can lead to significant visual impairment and, in severe cases, blindness [8,9,10]. Moreover, OT is one of the most prevalent infectious etiologies of posterior uveitis [7]. OT can lead to significant visual impairment and, in severe cases, complete blindness, with a propensity for recurrence [3].

This review aims to summarize and analyze existing research. It provides in-depth insights into the mechanisms of OT, explores genetic factors influencing susceptibility to infection, evaluates the efficacy of current diagnostic techniques, and discusses treatment strategies. We will explore the latest advancements in multimodal imaging techniques, including fundus fluorescein angiography (FFA), indocyanine green angiography (ICGA), optical coherence tomography (OCT), and optical coherence tomography angiography (OCTA), which have revolutionized the diagnosis and monitoring of OT. Additionally, we will examine the challenges in treating OT, including the limitations of current therapies and the high recurrence rates associated with the disease. This review will also highlight the geographical variations in OT prevalence and severity, emphasizing the need for tailored approaches in different regions. By comprehensively addressing these aspects, this study aims to contribute to a scientific understanding of *T. gondii* infection and inform clinical practice and public health policymaking, ultimately helping to reduce the risk of ocular complications and improve patients’ quality of life.

## 2. Overview of *Toxoplasma gondii*

### 2.1. Basic Biological Characteristics of Toxoplasma gondii

*T. gondii*, a protozoan parasite with a complex life cycle and multiple morphological stages, exhibits a unique host-specificity pattern [5,11]. Sexual reproduction occurs exclusively in the intestinal epithelium of felids (cats, the definitive hosts), resulting in the production and excretion of environmentally resistant oocysts. Asexual reproduction can occur in a wide range of warm-blooded animals, including humans (intermediate hosts), which become infected through ingestion of mature oocysts or tissue cysts in undercooked meat [5,11]. When intermediate hosts, such as humans, consume these oocysts, the sporozoites hatch within the gastrointestinal tract and release bradyzoites, thereby establishing a long-term latent infection. Upon reaching the eyes via the bloodstream, these parasites can form tissue cysts in the retina. Under certain conditions, such as immunosuppression, these cysts may rupture and lead to the release of tachyzoites [5,7].

The tachyzoites of *T. gondii* possess the ability to migrate to distant anatomical sites, including immune-privileged regions such as the placenta, brain, and eyes. Following invasion, the tachyzoites form a parasitophorous vacuole enveloped by a membrane. Through this vacuole, they secrete effector molecules that manipulate host cell metabolism and proliferate asexually via endodyogeny [6]. In instances where an effective immune response or therapeutic intervention is present, *T. gondii* can persist in tissues as bradyzoites, thereby establishing a chronic infection [11,12].

Bradyzoites enable the parasite to evade host immune surveillance and establish long-term latency within tissues [6]. The reversion of bradyzoites back to tachyzoites may be triggered by local or systemic immunosuppression, potentially leading to adverse consequences [5,7,11]. This cycle of latency and reactivation elucidates why OT symptoms often manifest with delayed onset, potentially emerging years after initial infection.

### 2.2. Strain Diversity of Toxoplasma gondii

*T. gondii* exhibits significant strain diversity, with three major lineages (Types I, II, and III) and various atypical strains identified [13,14]. These strains differ in virulence and epidemiological patterns, affecting disease outcomes and geographical distribution. In Europe, Type II strains dominate human infections, accounting for approximately 95% of cases. North America presents a more diverse landscape, with Type II strains representing 43.9% of human infections, Type III 18.2%, and atypical strains comprising the remainder [13,15]. The genetic diversity of *T. gondii* strains plays a crucial role in determining disease severity. South American *T. gondii* isolates exhibit greater genetic variability and typically higher virulence, contributing to more severe clinical manifestations in this region [13,14,16]. This geographical variation in strain distribution and virulence underscores the importance of molecular epidemiology in understanding toxoplasmosis patterns and outcomes.

### 2.3. Transmission Routes of Toxoplasma gondii

*T. gondii* utilizes multiple transmission routes, contributing to its widespread prevalence among humans and animals. The primary routes include foodborne transmission via consumption of undercooked meat containing tissue cysts or contaminated produce, environmental transmission via ingestion of oocysts from cat feces in soil or water, and congenital transmission from mother to fetus during pregnancy. Less common routes involve organ transplantation, blood transfusion, and laboratory accidents [5].

CT occurs when a pregnant woman is infected with *T. gondii* for the first time and subsequently transmits the parasite to her fetus. The risk of vertical transmission escalates with advancing gestational age; however, the severity of fetal involvement is inversely related to the timing of maternal infection [16]. Even in cases where infants present without overt symptoms at birth, CT can result in significant long-term consequences, with chorioretinitis being recognized as the most prevalent manifestation [14,16].

Felids, particularly domestic cats, play a critical role as definitive hosts in the life cycle of *T. gondii* [1,17]. Infected felines shed oocysts in their feces, which exhibit remarkable resilience and can survive for extended periods under various environmental conditions. This durability facilitates the widespread dissemination of *T. gondii* [11,17].

Oocyst contamination of water sources and soil may also lead to aerosolization, resulting in extensive environmental pollution. Such contamination pathways heighten the risk for humans and other animals to ingest oocysts through contaminated crops and water resources, contributing to several outbreaks of waterborne toxoplasmosis in humans [5,18].

## 3. Clinical Manifestation

### 3.1. Systemic Symptoms of Toxoplasmosis

Human toxoplasmosis presents in two main forms: congenital and acquired. The progression of the disease can be divided into three distinct phases: acute infection, characterized by the rapid proliferation of tachyzoites—the invasive form of *T. gondii*; latent infection, marked by the formation of tissue cysts containing bradyzoites; and reactivation, which is triggered by various factors, including immunosuppression. During this phase, bradyzoites revert to tachyzoites, resulting in a recurrence of acute disease [4].

In general, congenital infections are primarily induced by maternal infections acquired during pregnancy. The frequency of vertical transmission and the severity of fetal damage are contingent upon the gestational stage at which the maternal infection occurs. The susceptibility of the fetus to infection exhibits a positive correlation with gestational age, whereas the severity of the infection demonstrates a negative correlation with it [14,19]. When infections occur during the early stages of pregnancy, they can have profound effects on fetal development, often resulting in significant anomalies or miscarriage [11]. CT presents a broad spectrum of clinical manifestations, ranging from asymptomatic infections to severe multisystem disorders. The classic triad of CT includes ventricular dilatation, intracranial calcifications, and chorioretinitis [19]. The severity and prevalence of symptoms exhibit significant geographical variation, with particularly high rates of severe manifestations reported in South America. In this region, approximately 35% of CT-affected neonates present with severe neurological disorders, including hydrocephalus, microcephaly, and intellectual disability. Ocular involvement is observed in up to 80% of affected children, while hearing impairment affects as many as 40% [19,20,21]. This striking regional variation in disease severity has been attributed to differences in *T. gondii* genotypes, with more virulent strains predominating in South America [20,21].

In immunocompetent individuals, acute toxoplasmosis is typically self-limiting or asymptomatic. A minority of patients may present with flu-like symptoms, including fever, fatigue, myalgia, lymphadenopathy, and headache. These non-specific manifestations often lead to misdiagnosis as common viral infections [3,4]. It is noteworthy that, although chronic toxoplasmosis typically remains asymptomatic in individuals with normal immune function, persistent infections may be associated with various mental health disorders, such as schizophrenia and depression, as well as neurodegenerative diseases [22,23]. A meta-analysis published in 2023 compiled data from 49 studies involving 21,093 participants. It revealed an aggregated prevalence of chronic toxoplasmosis infection at 38.27% (95% CI: 32.04–44.9) among individuals with neuropsychiatric conditions, compared to 25.31% (95% CI: 21.53–29.08) in healthy controls, indicating a heterogeneity of 98.3% [22].

In patients with compromised immune function, such as those with HIV infection or those undergoing immunosuppressive therapy, toxoplasmosis often presents as a more severe and widespread systemic disease [24,25]. This may result from either new infections or the reactivation of previously latent infections. The most common and severe manifestation is the involvement of the central nervous system, typically presenting as toxoplasmic encephalitis [6]. In such cases, patients may experience symptoms including headaches, altered consciousness, seizures, and focal neurological deficits. If left untreated, this condition can lead to coma or even death [6]. While the central nervous system is a primary target, *T. gondii* exhibits a remarkable tropism for multiple organs. This multisystem involvement occurs either through direct parasitic encystment or secondary infection following dissemination from initial reactivation sites. After the brain, the lungs, eyes, and heart are most frequently affected, with cardiac involvement manifesting as myocarditis [6,11]. *T. gondii* has also been documented to be isolated from various tissues, including the liver, pancreas, skin, and bone marrow [11,26,27]. Notably, pulmonary or disseminated toxoplasmosis is particularly prevalent in transplant recipients, where it can lead to rapidly progressive infection and extensive parasitic dissemination [28,29].

### 3.2. Clinical Manifestation of Ocular Toxoplasmosis

OT can result from both congenital and acquired infections, with clinical presentations that often defy clear differentiation [19]. However, certain patterns emerge in clinical investigations that may suggest the origin of infection:

Congenital OT typically presents bilaterally, characterized by retinochoroidal scars, macular and posterior pole involvement, and a high degree of visual impairment despite less extensive inflammation. These cases often exhibit a high recurrence rate over years [3,30,31].

In contrast, acquired OT more frequently affects a single eye, rarely involves the macula, and is typically defined by extensive inflammation including retinal vasculitis, retinochoroiditis, and vitritis. Initial symptoms often include floaters and visual changes, which are usually reversible. Extraocular manifestations in acquired OT correlate with symptomatic Toxoplasma infection and vary depending on the host’s immune status [3,30,31].

Reactivation of latent *T. gondii* infection can occur at any time following primary infection, potentially due to the rupture of tissue cysts containing bradyzoites, which triggers a rapid local immune response. Acute OT typically manifests as a well-demarcated focus of retinal necrotizing inflammation, often accompanied by vitritis and diffuse inflammation in adjacent retinal and choroidal tissues [32]. This involvement of the underlying choroid, termed ‘retinochoroiditis’, characterizes the clinical picture. Active lesions appear as white foci with indistinct borders, frequently juxtaposed to atrophic or pigmented scars [30,32]. Vascular involvement is a common feature, with periphlebitis more prevalent than arteritis, often accompanied by retinal hemorrhages. In severe cases, vasculitis may extend to distant retinal areas [3,30]. A rare form of arteriolar inflammation, Kyrieleis arteritis, may be observed, manifesting as segmental, nodular, white intra-arterial plaques that do not extend beyond the vessel wall [30,33,34].

**Figure 1:**
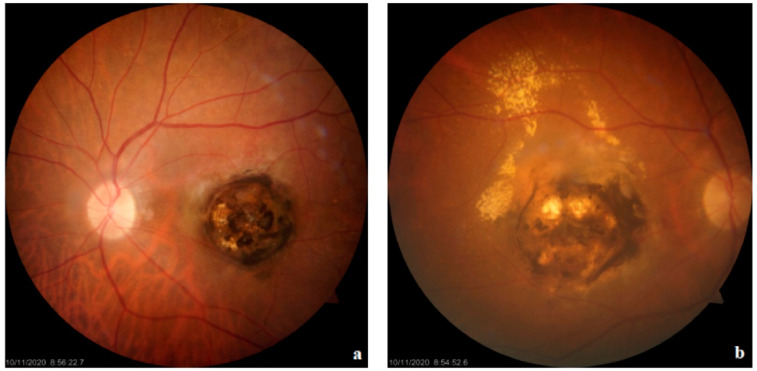
A 73-year-old woman diagnosed with chronic bilateral ocular toxoplasmosis presents with distinct findings in both eyes. (**a**) In the left eye (oculus sinister), a circular and well-defined macular retinochoroidal lesion is noted, characterized by irregularly scattered areas of atrophy and pigment deposition within the lesion. (**b**) In the right eye (oculus dexter), a macular retinochoroidal lesion is also observed, associated with choroidal neovascularization (CNV) and diffuse exudation. (Adapted with permission from Ref. [3]. 2022, Fabiani S et al.).

**Figure 2:**
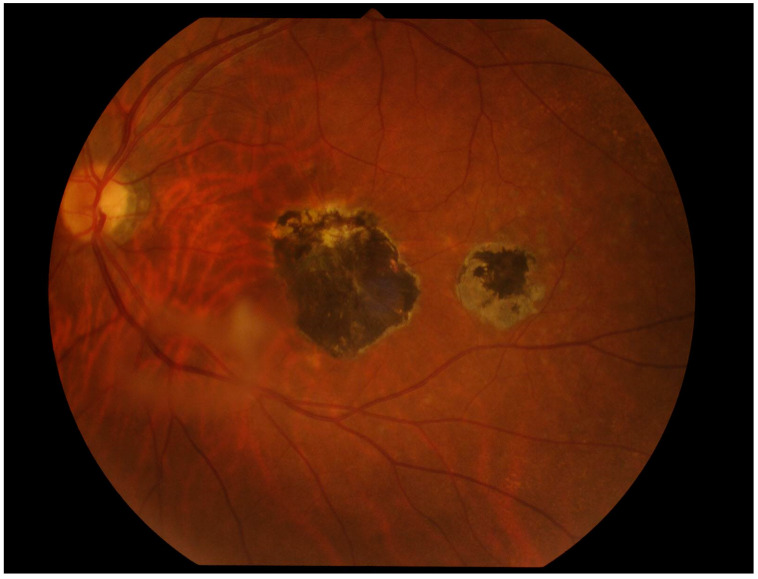
A 46-year-old man with ocular toxoplasmosis in left eye. The macula exhibits a necrotic scar lesion with a satellite lesion in close proximity.

OT in immunocompetent individuals often resolves spontaneously within 2 to 4 months. This healing process is marked by a centripetal regression of active lesions, resulting in an atrophic area that eventually transforms into a hyperpigmented scar due to disruption of the retinal pigment epithelium (see Figure 1 and Figure 2) [3,7,30,35]. However, the absence of posterior segment scarring is not pathognomonic; it neither excludes congenital toxoplasmosis nor definitively indicates recent infection [30].

OT can lead to a spectrum of complications, including secondary glaucoma, cataract formation, choroidal neovascularization (CNV), band keratopathy, scleritis, vascular occlusion, epiretinal membrane formation, cystoid macular edema, tractional retinal detachment, and optic nerve atrophy [3].

## 4. Diagnosis of Toxoplasma Infection

The diagnosis of *T. gondii* infection is a multifaceted process that employs various complementary methods. The diagnosis of active ocular inflammation in toxoplasmosis is typically established based on the distinctive findings observed in the posterior segment and is corroborated by serological testing. This typically presents as retinochoroiditis, characterized by focal necrotizing granulomatous retinitis, reactive granulomatous choroiditis, vitritis, and occasionally anterior segment inflammation [2,3,7]. This characteristic presentation often allows for clinical diagnosis without further diagnostic investigations. However, significant clinical variations can occur, posing diagnostic challenges. A thorough ocular examination is essential for diagnosing OT, as ophthalmologists can detect characteristic lesions such as retinitis, choroiditis, and optic neuritis. Techniques such as slit-lamp examination, fundoscopy, and fluorescein angiography are instrumental in identifying these manifestations [30].

### 4.1. Multimodal Imaging Techniques

Multimodal imaging techniques play a crucial role in the clinical diagnosis of OT. Table 1 summarized the multimodal imaging characteristics of OT at different clinical stages.

**Figure 3:**
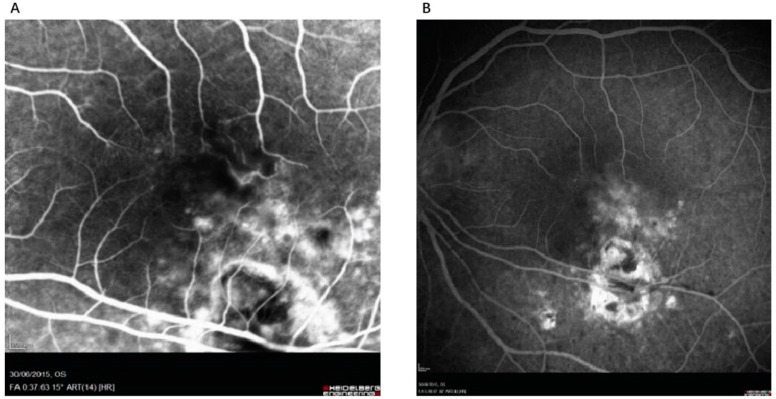
A 30-year-old woman, 6 months pregnant, presents with active recurrent toxoplasmic retinochoroiditis and its evolution following treatment. (**A**) The early phase of fundus fluorescein angiography (FFA) demonstrates progressive hyperfluorescence associated with the recurrent lesion. (**B**) The late phase of FFA reveals continued hyperfluorescence with centrifugal peripheral staining of the recurrent lesion. (Adapted from Ref. [37] 2020, Azar G et al.).

**Figure 4:**
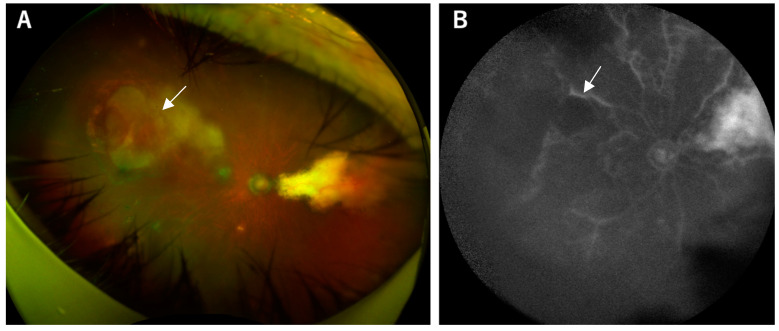
An 86-year-old woman with ocular toxoplasmosis (acquired infection) presents with significant findings in her right eye. (**A**) The color fundus image reveals a mixture of lesions and choroidal atrophy in the peripheral region of the right eye (oculus dexter). (**B**) Fluorescein angiography demonstrates characteristic features: In the early phase, the lesion exhibits hypofluorescence (black center) corresponding to the area of exudation (white arrow).

Fundus fluorescein angiography (FFA) serves as a key diagnostic tool for assessing acute and chronic ocular pathological vascular features. In acute toxoplasmic retinochoroiditis, FFA typically reveals initial hypofluorescence followed by variable progressive leakage at the lesion margins (Figure 3). Conversely, inactive lesions often exhibit hypofluorescence in early stages due to pigment hyperplasia blockage or hyperfluorescence in late stages due to retinal pigment epithelium atrophic changes (Figure 4) [36].

Indocyanine green angiography (ICGA), which detects ICG’s infrared fluorescence through the retinal pigment epithelium to visualize the choroidal vascular network, has played a pivotal role in elucidating the changes associated with acute OT [7]. This innovative technique has revealed significant choroidal involvement in cases of toxoplasmic retinochoroiditis. The predominant mechanism underlying this phenomenon is believed to be reactive inflammatory infiltration within the choroid, which leads to localized obstruction of choroidal vascular perfusion [36,38]. This condition manifests as focal areas of reduced indocyanine green fluorescence, with the affected regions often extending beyond the overlying active retinal lesions and correlating with localized choroidal thickening [7]. Research indicates that 75% of patients with acute toxoplasmic retinochoroiditis exhibit multiple low-fluorescence satellite dark dots, which tend to resolve following treatment [39]. Subsequent studies have demonstrated that in eyes with active toxoplasmic retinochoroiditis, the presence of multiple satellite dark dots is even more prevalent, reaching an incidence rate as high as 92% [38].

Optical coherence tomography (OCT) has emerged as a crucial tool in diagnosing and monitoring OT. This non-invasive, high-resolution imaging technique provides unprecedented insights into retinal and choroidal microstructures [40,41,42]. Recent advancements in spectral-domain OCT (SD-OCT), including enhanced depth imaging (EDI) and combined depth imaging (CDI), have significantly improved our ability to observe pathological changes in toxoplasmic retinochoroiditis [36]. The advent of swept-source OCT (SS-OCT) has been particularly noteworthy, offering simultaneous visualization of choroidal, retinal, and vitreous alterations in a single scan, thereby providing more comprehensive clinical information [43].

**Figure 5:**
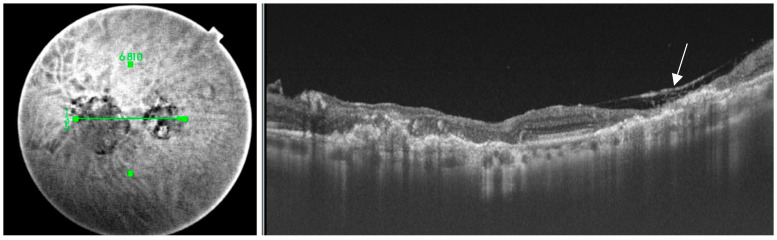
The left image shows the scan line across a toxoplasmosis lesion, The right image demonstrates a reduction in retinal thickness (white arrow), which is indicative of tissue atrophy in the affected area.

OCT demonstrates unique advantages in diagnosing OT, assessing disease extent and severity, and tracking real-time changes. Typical OCT findings include increased intraretinal reflectivity, disorganization of retinal layers, and choroidal shadowing [44]. In addition, OCT can be a valuable tool in detecting subtle pathological changes, particularly in cases where a pigmented retinal scar is not readily apparent [45]. Observational studies have further demonstrated that OCT is particularly effective in differentiating OT based on the disease’s activity [46]. Active lesions show increased retinal thickness and reflectivity with blurred layer details, often accompanied by choroidal granulomas and serous retinal detachment. Partially active lesions exhibit posterior hyaloid thickening and epiretinal membrane formation. Inactive lesions demonstrate reduced retinal thickness, as shown in Figure 5, indicating tissue atrophy in the affected area. A characteristic “hourglass configuration”, featuring RPE changes and retina-RPE approximation, is observed in both partially active and inactive stages, indicating decreased lesion activity [46]. These OCT features aid in assessing disease progression and guiding clinical management.

**Figure 6:**
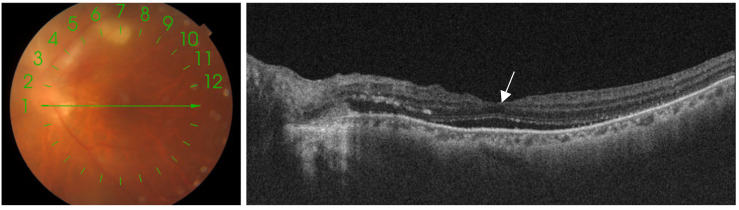
The left image shows the scan line across a toxoplasmosis lesion, The right image displays serous retinal detachment in the macular region (white arrow).

OCT can reveal both inner and outer retinal changes in patients with punctate outer retinal toxoplasmosis (PORT), an uncommon presentation of OT, including disruptions in the retinal pigment epithelium/Bruch’s membrane complex, the ellipsoid zone, and the interdigitation zone. Additionally, OCT can detect hyperreflective, punctate, and pre-retinal lesions at the vitreoretinal interface [36,47]. These findings aid in differentiating PORT from other causes of white dot syndromes or unilateral retinitis [47]. Moreover, OCT serves as a useful instrument for the surveillance of complications associated with OT. These complications encompass a range of conditions, including epiretinal membranes, serous retinal detachment, vitreomacular traction, macular holes, outer retinal tubulation, cystoid macular edema, and choroidal neovascularization [36,48,49] (Figure 6).

Optical Coherence Tomography Angiography (OCTA) generates high-resolution retinal and choroidal microvascular flow maps by analyzing variations in signal characteristics between consecutive OCT scans [50]. This includes assessments of light reflectance intensity, phase shifts, and phase variance. OCTA is capable of visualizing the microvascular architecture of the retina and choroid with a resolution on the order of micrometers, all while eliminating the need for contrast agent injections. Consequently, it circumvents the potential adverse effects associated with traditional fluorescein angiography [50]. Observational studies have revealed that OCTA offers significant insights into the vascular alterations associated with toxoplasmic retinochoroiditis at various stages of the disease. Active lesions typically show increased flow signal in inner retinal layers, decreased flow in outer retina and choriocapillaris, and potential abnormal vascular networks. As lesions progress to partial activity and inactivity, there is a gradual reduction in flow signal across all retinal layers and persistent decreased flow in the choriocapillaris. Chronic or scarred lesions exhibit marked reduction or absence of flow signal in all layers within the affected area [37]. These OCTA findings complement OCT, aiding in the assessment of disease activity, extent of vascular involvement, and treatment response. This non-invasive imaging modality enhances early diagnosis and management of complications such as choroidal neovascularization in toxoplasmic retinochoroiditis.

### 4.2. Laboratory Diagnosis

Laboratory diagnosis of toxoplasmosis relies on two main approaches: indirect methods, which detect specific antibodies of various isotypes, and direct methods, which identify parasites or their DNA in clinical specimens [6,51]. Table 2 summarizes the diagnostic strategies employed for OT across various patient populations.

In immunocompetent individuals, the diagnosis of *T. gondii* infection primarily relies on serological testing, detecting specific antibodies such as IgG and IgM, which indicate current or past infection. Serological screening is particularly crucial in three scenarios: early pregnancy, patients with retinochoroiditis without known congenital infection history, AIDS-positive patients, and transplantation settings, for both organ donors and recipients [52]. Furthermore, serological testing serves as a principal diagnostic tool in patients presenting with lymphadenopathy or fever, facilitating differential diagnosis from infections such as Epstein–Barr virus, cytomegalovirus, or HIV, as well as other lymphadenopathy-associated conditions, including hematological malignancies [6,53,54,55]. In cases of Toxoplasma infection, IgM antibodies are typically the first to emerge, becoming detectable within one week post-infection. Their levels increase and usually reach a peak within one to three months. Subsequently, in the majority of patients (over 70%), IgM levels gradually decline over the course of the following nine months until they ultimately become negative [52]. Toxoplasma-specific IgG, on the other hand, emerges around 2 weeks post-infection, reaching peak levels at approximately 3 months. It then maintains a plateau for about 6 months before slowly declining to lower levels, persisting throughout the infected individual’s lifetime due to the presence of latent cysts in immune-privileged organs [52]. Furthermore, although not commonly tested, the production kinetics of IgA antibodies mirror those of IgM, with IgA peaking at a later time and persisting for 3 to 4 months post-infection [52,56]. It is important to recognize that serological testing is not without its limitations, as false-positive and false-negative results can occur, particularly in immunocompromised patients. False positives may result from cross-reactivity with structures of similar pathogens, laboratory errors, or the presence of residual antibodies from previous infections. Conversely, false negatives are often encountered during the early stages of infection or in immunosuppressed individuals who exhibit inadequate immune responses. Ophthalmologists should note that patients with OT often exhibit low levels of Toxoplasma-specific IgG antibodies and negative IgM serum titers [2]. Given the high prevalence of seropositive cases in certain populations, a positive IgG result alone is generally considered insufficient for a definitive diagnosis of OT [2,52].

In cases of uncertain diagnosis, ocular tissues and fluids should be obtained for protozoal parasite detection through polymerase chain reaction (PCR) and/or serological testing. While PCR offers high sensitivity and specificity, serological tests, particularly the enzyme-linked immunosorbent assay (ELISA), are considered the ‘gold standard’ for laboratory confirmation of OT in clinical settings [6,51,57]. However, due to the limited volume of samples obtained from the eye and the compromise of the blood-retinal barrier, the sensitivity and specificity for detecting intraocular specific antibodies in immunocompetent individuals are 63% and 89%, respectively [2]. Conversely, in immunocompromised patients, the production of ocular antibodies is often unpredictable [2,57]. A positive PCR result can confirm a diagnosis; however, if results yield negative findings, one can evaluate the ratio of specific IgG to total IgG in aqueous humor and serum by calculating the Goldmann–Witmer coefficient (GWC). A GWC greater than 1 typically indicates the presence of specific antibody production within the eye; nonetheless, such results may also occur in healthy individuals. Consequently, a GWC exceeding 2 or at least 3 is required to be deemed positive for definitive diagnosis [2,58]. The sensitivity of GWC is approximately 50%, with rates of 57% in immunocompromised patients and 93% in those with normal immune function [2,30].

If GWC fails to distinguish between locally produced antibodies and systemic antibodies, Western blot (WB) analysis can be performed on concurrently collected serum and aqueous humor samples. The sensitivity and specificity of WB analysis (>95%) surpass that of GWC and are less affected by disruptions to the blood-retinal barrier. However, sensitivity may be influenced by the interval between symptom onset and sample collection [2,52]. Mathis et al. conducted a retrospective analysis of 42 patients with suspected OT and 45 patients with other suspected ocular inflammatory diseases who underwent consecutive aqueous humor and serum examinations [59]. The study aimed to compare the sensitivity of WB and GWC tests. Results demonstrated that in aqueous humor samples collected within the first three weeks, WB exhibited significantly higher sensitivity compared to GWC (64.7% vs. 23.5%, *p* = 0.039). However, after three weeks, no significant difference in sensitivity was observed between the two methods (76% vs. 64%, *p* = 0.625) [59].

The integration of PCR, GWC, and WB techniques can significantly enhance both the sensitivity and specificity in diagnosing OT. When used in combination, these methods can achieve a sensitivity of up to 97% [2,60]. Furthermore, measurement of IgM and IgA antibodies in aqueous humor serves as a valuable indicator for acute phase diagnosis and monitoring treatment response [30,57,60].

## 5. Treatment

Toxoplasma-associated chorioretinitis in immunocompetent patients is typically self-limiting, resolving within 4–8 weeks [3,61]. The treatment of OT remains controversial due to the disease’s natural course and potential drug side effects. While some clinicians opt not to treat small peripheral retinal lesions, others treat all patients to reduce recurrence and complication rates. Treatment decisions for OT require consideration of infection severity, patient immune status, and potential complications [2].

Antiparasitic drugs form the cornerstone of treatment, with pyrimethamine and sulfadiazine being commonly used. This combination inhibits parasite folate synthesis, thereby preventing nucleic acid synthesis and replication, ultimately suppressing *T. gondii* proliferation and reducing intraocular inflammation [61]. While pyrimethamine and sulfadiazine remain the cornerstone therapies for OT, it is essential to conduct vigilant monitoring due to the possibility of adverse events. These include hematological abnormalities (leukopenia, thrombocytopenia), gastrointestinal disturbances, and dermatological reactions [2]. To mitigate these risks, weekly complete blood count monitoring is imperative throughout the treatment course. Furthermore, concomitant folinic acid supplementation is essential to counteract the antifolate effects of pyrimethamine, thereby reducing the risk of bone marrow suppression [2,7,61].

In cases of excessive immune response leading to additional tissue damage or disease exacerbation, corticosteroids or other immunosuppressants may be necessary to modulate immune response intensity and prevent further ocular tissue damage. The classic “triple therapy” includes pyrimethamine, sulfadiazine, and systemic corticosteroids (most commonly prednisone) [61]. Notably, in the absence of concurrent antiparasitic medication, the administration of corticosteroids is significantly more likely to result in adverse outcomes [30].

Trimethoprim-sulfamethoxazole (TMP-SMX) is another important antibiotic combination used in the treatment of OT. The rationale behind the use of TMP-SMX is that trimethoprim inhibits the synthesis of tetrahydrofolate, while sulfamethoxazole blocks the utilization of para-aminobenzoic acid, another essential component for the parasite’s growth [62,63]. One of the advantages of TMP-SMX is its relatively good ocular penetration and bioavailability, which allows the drugs to reach therapeutic concentrations in the eye [64]. Additionally, TMP-SMX is generally well-tolerated, with the most common side effects being gastrointestinal disturbances and skin rashes [64]. Accumulating evidence demonstrates that TMP-SMX, either as monotherapy or in combination with corticosteroids, effectively mitigates the inflammatory response, enhances visual outcomes, and reduces recurrence rates in patients with OT [64,65,66,67]. Furthermore, studies have shown that the use of TMP-SMX as a maintenance or suppressive therapy can significantly reduce the risk of recurrence in patients with a history of frequent and/or severe relapses of Toxoplasma retinochoroiditis [68,69]. This adjunctive prophylactic treatment with TMP-SMX may be particularly beneficial for select individuals who have demonstrated a propensity for recurrent OT episodes. However, it is important to note that some patients may develop hypersensitivity reactions or experience more severe adverse effects, such as Stevens–Johnson syndrome or toxic epidermal necrolysis, with TMP-SMX therapy [58]. In such cases, alternative antibiotic combinations or regimens may need to be considered.

Clindamycin, a lincosamide antibiotic that concentrates in ocular tissues and penetrates tissue cyst walls, can be added to the classic triple therapy as a “quadruple therapy”. This combination can effectively suppress the proliferation of *T. gondii*. Research has shown that the use of clindamycin alone can achieve relatively good therapeutic effects, but it is usually used in combination with other anti-Toxoplasma drugs such as pyrimethamine, sulfadiazine, or trimethoprim-sulfamethoxazole to enhance the efficacy, shorten the treatment course, and reduce the risk of recurrence [67]. Additionally, clindamycin can also help reduce the inflammatory response in the retina caused by Toxoplasma infection, thereby improving visual prognosis [2,67]. The most common adverse effects associated with clindamycin include gastrointestinal symptoms such as nausea, vomiting, and diarrhea. In rare cases, more serious side effects such as pseudomembranous colitis, a potentially life-threatening condition caused by Clostridioides difficile infection, can occur [70]. Clinicians should closely monitor patients for the development of any adverse reactions during clindamycin therapy. If severe gastrointestinal symptoms or other serious side effects emerge, the medication should be discontinued immediately [70,71]. Discontinuation of clindamycin may also be warranted in the event of severe allergic reactions, such as Drug Reaction with Eosinophilia and Systemic Symptoms (DRESS) or Stevens–Johnson syndrome [71].

In recent years, alternative treatment modalities have emerged for the management of OT, complementing standard systemic or localized (intravitreal) antibiotic regimens. One such approach that has demonstrated efficacy is the intravitreal administration of clindamycin combined with dexamethasone. Clinical studies have shown that this targeted, localized treatment can effectively control Toxoplasma retinochoroiditis [72].

Overall, while current therapeutic approaches, including antimicrobial agents and immunosuppressants, can control active infections, they neither provide a definitive cure nor completely eliminate the risk of recurrence. The definition of clinical endpoints remains controversial, with treatment goals potentially varying among patients. Moreover, existing therapies may induce adverse effects, necessitating a careful balance between efficacy and safety.

## 6. Recurrence Patterns and Risk Factors in Ocular Toxoplasmosis

OT remains a significant challenge in ophthalmology due to its high recurrence rate. A long-term study conducted by Bosch-Driessen et al. has revealed that a substantial proportion of patients experience reactivation, with estimates ranging from 60% to 86% depending on the definition of recurrence and follow-up duration [30].

The temporal pattern of recurrences is noteworthy. In the previously mentioned cohort study, approximately 54% of recurrences occur within the first two years post-episode. More specifically, 64.3% of new recurrences occur within the first year, followed by 21.4% in the second year and 14.3% in the third year [30]. This pattern underscores the critical need for intensive monitoring during the early post-episode phase.

Striking geographical variations in OT prevalence and severity have been observed. In Brazil, up to 50–80% of women of childbearing potential and 50% of primary school students test positive for antibodies to *T. gondii*. South American countries report not only higher incidence rates but also more aggressive disease patterns, attributed to the predominance of highly virulent *T. gondii* strains (types I, III, and atypical) in these areas [73,74].

Treatment history and prophylactic strategies play crucial roles in managing ocular toxoplasmosis recurrence. A Colombian study by de-la-Torre et al. identified previous therapy with steroids without antibiotics and subconjunctival steroid injections as significant risk factors for recurrence, highlighting the importance of appropriate antibiotic coverage [75]. In response to these challenges, long-term antibiotic prophylaxis has emerged as a promising approach. Silveira et al.’s study in Brazil demonstrated a significant reduction in recurrence rates (6.6% vs. 23.8% in the control group) with trimethoprim/sulfamethoxazole prophylaxis [69]. This finding was further corroborated by Felix et al.’s clinical trial, which showed a dramatic reduction in cumulative recurrence probability over three years (0% vs. 20.3% in the placebo group, *p* < 0.001) [76]. Alternative treatment regimens have also been explored, with Bosch-Driessen et al.’s study showing comparable efficacy and better tolerability when replacing sulfadiazine with azithromycin in combination with pyrimethamine [77]. Similarly, Soheilian et al. found trimethoprim/sulfamethoxazole to be as effective as the classic pyrimethamine/sulfadiazine treatment, with comparable recurrence rates (10.16% vs. 10.16%) [78]. These findings collectively underscore the importance of tailored prophylactic and treatment strategies in managing ocular toxoplasmosis, paving the way for more effective and patient-friendly approaches to prevent recurrence.

The relationship between pregnancy and OT recurrence has been a subject of debate in recent years. Several studies have investigated this association, yielding conflicting results. In the Netherlands, Braakenburg et al. conducted a retrospective longitudinal cohort study involving 50 women of childbearing age [79]. Their findings suggested that recurrence rates of OT were not higher during pregnancy, with incident rate ratios for pregnant versus non-pregnant intervals indicating lower recurrence rates during pregnancy (IRR 0.54–0.75, *p* > 0.05) [79]. Similarly, a study conducted by Reich M et al. in Germany compared 17 pregnant women diagnosed with OT to 29 non-pregnant controls, revealing no evidence to suggest an increased recurrence rate during pregnancy [80]. In fact, they observed a lower average annual recurrence rate during pregnancy (0.16) compared to non-pregnant individuals (0.32; *p* = 0.088). Furthermore, the average annual recurrence rate in the control group (0.31) was significantly higher than that of the cases during pregnancy (*p* = 0.002) [80]. Conversely, research from Poland by Brydak-Godowska J et al. reported contrasting results [81]. Their retrospective analysis of 213 women of childbearing age demonstrated a 7.4-fold higher risk of toxoplasmic retinochoroiditis recurrence during pregnancy compared to non-pregnant periods (*p* < 0.0001) [81]. These divergent findings highlight the complexity of the relationship between pregnancy and ocular toxoplasmosis recurrence, emphasizing the need for further research to elucidate the underlying mechanisms and provide clear clinical guidance for managing ocular toxoplasmosis in pregnant women.

Patient-Specific Risk Factors are listed below:

1. Age: Age-related factors significantly influence recurrence patterns in ocular toxoplasmosis. Younger individuals, particularly those under 40, exhibit a higher risk of recurrence, possibly due to more robust immune responses or hormonal influences [82]. Patients with congenital toxoplasmosis face an elevated lifelong risk, with more frequent recurrences during childhood and adolescence [83]. While recurrence risk generally decreases with age, older adults may experience complications arising from age-related changes in ocular structures and immune function [82].
2. Immune Status: Immunocompromised patients face a heightened risk of disease reactivation [7].
3. Pregnancy: Bosch-Driessen et al. documented that among the 82 female patients diagnosed with OT, seven (constituting 9%) experienced recurrences of the ocular condition during pregnancy, with four individuals exhibiting this recurrence in each subsequent gestation [30].
4. Ocular Characteristics: Patients with bilateral retinal scars at initial presentation have a higher risk of developing recurrent disease in both eyes. Contralateral eyes with subclinical retinal scars show a 40% recurrence rate compared to 4% in unaffected contralateral eyes (*p* < 0.001) [30].
5. COVID-19 infection: Recent case reports have documented OT reactivation following both COVID-19 infection and vaccination, highlighting the need for vigilance in OT patients during the ongoing pandemic [84,85].

## 7. Conclusions

*T. gondii* is a ubiquitous parasite with a complex life cycle and diverse transmission routes, posing significant public health challenges worldwide. Ocular toxoplasmosis, a severe manifestation of *T. gondii* infection, can lead to potentially blinding complications. The clinical presentation of OT varies, necessitating a comprehensive diagnostic approach that combines clinical examination, multimodal imaging techniques (including FFA, ICGA, OCT, and OCTA), and laboratory tests. These advanced imaging modalities have significantly improved our ability to diagnose, monitor, and manage OT. However, the specific mechanisms underlying ocular involvement remain unclear, and current diagnostic options have limitations. Future research should focus on elucidating the pathogenesis of OT and developing more sensitive and specific diagnostic tools. The treatment of OT remains challenging, with current therapies unable to provide a definitive cure or completely prevent recurrence. The high recurrence rates, particularly in certain geographical regions and patient populations, underscore the need for improved therapeutic strategies and long-term management approaches.

In conclusion, this comprehensive review highlights the complex nature of *T. gondii* infection and its ocular manifestations, emphasizing the need for continued research and collaborative efforts to improve diagnosis, treatment, and prevention of ocular toxoplasmosis. By addressing these challenges, we can work towards reducing the global burden of this potentially sight-threatening condition and improving the quality of life for affected individuals.

## Figures and Tables

**Table 1:** Multimodal imaging characteristics of ocular toxoplasmosis at different clinical stages.

Clinical Stage	Fundus Photography Features	Fundus Autofluorescence (FAF) Features	Fluorescein Angiography (FFA) Features	Indocyanine Green Angiography (ICGA) Features	Optical Coherence Tomography (OCT) Features	Optical Coherence Tomography Angiography (OCTA) Features	Key Diagnostic Points
Active Lesion	Gray-white necrotic foci in posterior pole with fuzzy borders; surrounding inflammation and hemorrhage	Subtle hyper/isoautofluorescence; thickening and detachment of posterior hyaloid over the lesion with irregular hyperreflective deposits	Early hypofluorescence, late progressive leakage at lesion margins; possible optic nerve head hyperfluorescence, vessel leakage, and vascular sheathing	Hypofluorescence; multiple satellite dark dots extending beyond the active retinal lesion; localized choroidal hypoperfusion	Increased intraretinal reflectivity with disorganization of neuroretinal layer boundaries corresponding to the area of retinitis; thickened and hyporeflective choroid beneath the active lesion; posterior hyaloid thickening with irregular hyperreflective deposits over the lesion	Hyporeflectivity of the lesion with diffuse choroidal dilation and many collateral vascular branches surrounding; decreased flow in outer retina and choriocapillaris	Fuzzy lesion margins, surrounding retinal edema; “headlight in the fog” appearance due to vitritis; ICGA shows multiple satellite dark dots
Inactive/Scar Lesion	Well-defined pigmented scar with sharp margins, often with central atrophy	Hypoautofluorescent	Early hypofluorescence due to pigment blockage or window defect, late staining at margins	Hypofluorescence; disappearance of satellite dark dots	Disorganized retinal layer reflectivity beneath a thin, hyperreflective choroid; RPE-choroid complex atrophy; persistent “hourglass configuration” with RPE changes and retina-RPE approximation	Marked reduction or absence of flow signal in all layers within the affected area	Clear boundaries, no signs of active inflammation; OCT shows tissue atrophy and “hourglass configuration”
Recurrent Lesion	New active lesion typically adjacent to old inactive pigmented scar (satellite lesion)	Mixture of hyper/isoautofluorescence (new lesion) and hypoautofluorescence (old scar)	Early hypofluorescence in new lesion area, late hyperfluorescence with leakage	Hypofluorescence; reappearance of satellite dark dots around new lesion	New area of retinal thickening adjacent to old atrophic area; posterior hyaloid thickening and epiretinal membrane formation	Changes in flow signal in new lesion area: increased in inner retina, decreased in outer retina and choriocapillaris	Coexistence of new active lesion and old scar; OCT shows both acute and chronic changes
Congenital Lesion	“Horseshoe” or ring-shaped macular scar	Hypoautofluorescent scar	Hypofluorescence in scar area	Hypofluorescence in scar area; possible choroidal filling defects	Macular retinal atrophy, disorganized retinal structure, possible outer retinal tubulation	Reduced or absent flow in all retinal layers and choriocapillaris in the scar area	Typical macular scar morphology; severe visual impairment from birth

**Table 2:** Diagnostic strategies for ocular toxoplasmosis in different patient populations.

Patient Type	Disease Setting	Laboratory Diagnostic Approach	
		Diagnostic Modality	Specific Tests
Immunocompetent	Primary infection or reactivation	Serological testing	IgG/IgM antibody detection by ELISA, immunoblot
		Aqueous/vitreous sampling	PCR, antigen detection, Toxoplasma-specific antibody testing by immunoblot
Pregnant Women	Maternal primary infection	Serological testing	IgG/IgM antibody detection by ELISA, immunoblot to determine time of infection
		Amniotic fluid testing	PCR, antigen detection
Neonates	Congenital infection	Serological testing	IgG/IgM antibody detection by ELISA, immunoblot
		CSF/Tissue testing	PCR, antigen detection, histopathology
Immunocompromised	Reactivation or disseminated infection	Serological testing	IgG/IgM antibody detection by ELISA, immunoblot (may be false negative)
		Aqueous/vitreous sampling	PCR, antigen detection, Toxoplasma-specific antibody testing by immunoblot
		CSF/Tissue testing	PCR, antigen detection, histopathology
Fetus	Congenital infection	Amniotic fluid testing	PCR, antigen detection

Abbreviations: ELISA: Enzyme-Linked Immunosorbent Assay; PCR: Polymerase Chain Reaction; CSF: Cerebrospinal Fluid.
